# Health worker adherence to malaria treatment guidelines at outpatient health facilities in southern Malawi following implementation of universal access to diagnostic testing

**DOI:** 10.1186/s12936-017-1693-3

**Published:** 2017-01-23

**Authors:** Ruth J. Namuyinga, Dyson Mwandama, Dubulao Moyo, Austin Gumbo, Peter Troell, Miwako Kobayashi, Monica Shah, Andrew Bauleni, Jodi Vanden Eng, Alexander K. Rowe, Don P. Mathanga, Laura C. Steinhardt

**Affiliations:** 1 Malaria Branch, Division of Parasitic Diseases and Malaria, Centers for Disease Control and Prevention, Atlanta, GA USA; 2 Malaria Alert Centre, College of Medicine, University of Malawi, Blantyre, Malawi; 3 National Malaria Control Programme, Ministry of Health, Lilongwe, Malawi; 4 Malaria Branch, Division of Parasitic Diseases and Malaria, Centers for Disease Control and Prevention, Lilongwe, Malawi; 5 National Center for Immunization and Respiratory Diseases, Division of Bacterial Diseases, Centers for Disease Control and Prevention, Atlanta, GA USA; 6 Global Immunization Division, Centers for Disease Control and Prevention, Atlanta, GA USA

**Keywords:** Malaria, Case management, Guidelines, Outpatient, Health worker, Adherence, Testing, Treatment, Malawi

## Abstract

**Background:**

Appropriate diagnosis and treatment are essential for reducing malaria mortality. A cross-sectional outpatient health facility (HF) survey was conducted in southern Malawi from January to March 2015 to determine appropriate malaria testing and treatment practices four years after implementation of a policy requiring diagnostic confirmation before treatment.

**Methods:**

Enrolled patients were interviewed, examined and had their health booklet reviewed. Health workers (HWs) were asked about training, supervision and access to the 2013 national malaria treatment guidelines. HFs were assessed for malaria diagnostic and treatment capacity. Weighted descriptive analyses and logistic regression of patient, HW and HF characteristics related to testing and treatment were performed.

**Results:**

An evaluation of 105 HFs, and interviews of 150 HWs and 2342 patients was completed. Of 1427 suspect uncomplicated malaria patients seen at HFs with testing available, 1072 (75.7%) were tested, and 547 (53.2%) tested positive. Testing was more likely if patients spontaneously reported fever (odds ratio (OR) 2.6; 95% confidence interval (CI) 1.7–4.0), headache (OR 1.5; 95% CI 1.1–2.1) or vomiting (OR 2.0; 95% CI 1.0–4.0) to HWs and less likely if they reported skin problems (OR 0.4; 95% CI 0.2–0.6). Altogether, 511 (92.7%) confirmed cases and 98 (60.3%) of 178 presumed uncomplicated malaria patients (at HFs without testing) were appropriately treated, while 500 (96.6%) of 525 patients with negative tests did not receive anti-malarials. Only eight (5.7%) suspect severe malaria patients received appropriate pre-referral treatment. Appropriate treatment was more likely for presumed uncomplicated malaria patients (at HFs without testing) with elevated temperature (OR 1.5/1 °C increase; 95% CI 1.1–1.9), who reported fever to HWs (OR 5.7; 95% CI 1.9–17.6), were seen by HWs with additional supervision visits in the previous 6 months (OR 1.2/additional visit; 95% CI 1.0–1.4), or were seen by older HWs (OR 1.1/year of age; 95% CI 1.0–1.1).

**Conclusions:**

Correct testing and treatment practices were reasonably good for uncomplicated malaria when testing was available. Pre-referral treatment for suspect severe malaria was unacceptably rare. Encouraging HWs to elicit and appropriately respond to patient symptoms may improve practices.

## Background

Approximately 214 million malaria cases and 438,000 malaria deaths occurred worldwide in 2015 [1]. Use of artemisinin-based combination therapy (ACT) for uncomplicated malaria and parenteral quinine or artemisinins for severe malaria is highly effective in preventing malaria deaths when prompt diagnosis and timely treatment is initiated [2]. Since 2010, to better target anti-malarial treatment and more appropriately manage non-malarial fevers, the World Health Organization (WHO) has recommended diagnostic testing of all suspect malaria patients before initiating treatment [3]. Nearly all malaria-endemic countries have updated their malaria case-management policies to reflect these recommendations and most have made rapid diagnostic tests (RDTs) for malaria widely available at health facilities (HFs). However, gaps in health worker (HW) practices based on current recommendations have been reported in a variety of settings. A recent systematic review and meta-analysis of 14 studies, 11 of which were conducted after 2010, reported that administration of appropriate malaria treatment based on RDT results ranged from 39.7% in Zambia to 99.9% in Zanzibar [4].

The entire population of Malawi (18 million people) is at risk for malaria [5]. An estimated six million cases of malaria occur annually, accounting for 29% of outpatient visits across all ages and 40% of all hospitalizations among children under five years of age. Presumptive treatment of malaria among febrile patients was the norm until 2011, when the Malawi National Malaria Control Programme (NMCP) adopted the WHO recommendation to diagnostically confirm malaria cases before initiating treatment [6]. To support this initiative, the Malawi Ministry of Health (MoH) made RDTs widely available in HFs across the country later that year. Prompt and effective case management of malaria patients is one of the key malaria control efforts in Malawi [6], but previous studies have indicated shortcomings in correct treatment. Prior to the widespread availability of RDTs in Malawi, a 2011 national HF survey reported that only 67% of patients with uncomplicated malaria received a prescription for the first-line ACT, artemether-lumefantrine (AL) [7]. In addition, 31% of patients without malaria received AL [7], and 22% of patients with a negative microscopy test at the HF were prescribed AL [8].

To promote HWs’ knowledge and adherence to national malaria treatment guidelines, the NMCP has utilized a multipronged approach, including provision of in-service and refresher training, distribution of printed copies of the guidelines and periodic supportive supervision. In 2014 and 2015, HWs in Malawi received refresher training on malaria case management based on the latest malaria treatment guidelines released in July 2013. In addition to recommending testing of suspect uncomplicated malaria patients before administration of ACT, the guidelines emphasized immediate administration of rectal (primarily at community level) or parenteral artesunate or parenteral quinine as pre-referral, life-saving treatment for severe malaria at outpatient HFs [9].

The objective of this survey was to assess the quality of malaria case management at outpatient HFs, based on HW adherence to Malawi’s latest national malaria treatment guidelines, almost 4 years after roll-out of RDTs and several months after malaria case-management refresher training.

In addition, an evaluation of HF, HW and patient-level attributes and their associations with appropriate testing and treatment of suspect uncomplicated malaria patients was performed to identify opportunities to improve case-management quality.

## Methods

### Study design and data collection

This cross-sectional study was conducted in southern Malawi, a region with malaria prevalence estimates among children under 5 years old of 30% [6] and among 5 to 21 years olds of 60% [10]. The survey collected baseline data for a cluster-randomized, controlled trial to evaluate the effectiveness of mobile phone text-message reminders to HWs to improve the case management of fever, pneumonia and diarrhoea. Seven of 13 districts in the region were selected if their HWs had received malaria case-management refresher training in 2014. The seven districts were: Chikwawa, Nsanje, Thyolo, Blantyre, Chiradzulu, Mulanje, and Phalombe. The majority (89%) of HFs in Malawi are operated by either the MoH or Christian Health Association of Malawi (CHAM) [11], and all such HFs follow the national malaria treatment guidelines. MoH facilities provide free services, while CHAM facilities require a small co-pay. The survey was performed to provide baseline data for a cluster-randomized controlled trial of text message reminders to HWs to assess their impact on HW adherence to national malaria treatment guidelines.

Only health centres and district, community or rural hospitals operated by the MoH or CHAM with a functional outpatient department (OPD), and road accessibility were included. The targeted sample size was 105 facilities based on sample sizes needed for the larger text message trial. From 22 January to 5 March, 2015, during high malaria transmission season, survey teams evaluated the 105 participating facilities and assessed HW adherence to the latest national malaria treatment guidelines. During the survey, six facilities in Chikwawa and Nsanje could not be accessed due to poor road conditions during the rainy season and were, therefore, replaced by six facilities from Phalombe district.

Data were collected by four survey teams via questionnaires in CommCare version 2.24.0 (Dimagi, Inc) using ASUS Nexus 7 Android tablets (Taipei, Taiwan). Each team visited one HF for a full day during the working week (Monday to Friday) and randomly selected one OPD (if the facility had more than one) and conducted patient exit interviews, HW interviews and HF assessments.

All patients attending selected OPDs between 08:00 and 16:00 h on the day of the survey comprised the sampling frame. Patients were screened for eligibility after selection using systematic sampling with a skip interval determined by patient volume 1 week prior, calculated to yield approximately 20 patients/HF day. The first patient within the skip interval was randomly selected. If a selected patient did not meet eligibility criteria, investigators approached the next patient using the pre-determined skip interval. Selected patients were enrolled if they met eligibility criteria which were that it was their first visit to the facility for the current illness and they or their caregivers provided written informed consent. All HWs performing clinical consultations in the selected OPDs were eligible for interviews and were assigned unique identification numbers. Enrolled patients were given study cards on which HWs placed their identification numbers and recorded patient diagnoses during the clinical encounter.

Patient exit interviews were completed at the end of the patient’s visit. Surveyors asked patients (or their caregivers) about current malaria-related symptoms spontaneously reported to the HW, whether HWs had specifically asked about the presence/history of fever, any anti-malarials they had taken at home, their clinical encounter with the HW, any laboratory tests done, and any anti-malarial medications administered, dispensed or prescribed at the HF. If ACT were dispensed to the patients, their knowledge regarding the number of ACT tablets to take for each dose, the number of doses to take per day, and the number of days to take the entire ACT course was evaluated through open-ended questions. Additional patient information was obtained from the patient’s personal health booklet, the individual health record on which their clinical encounter is typically documented at the HF. Surveyors then conducted a brief physical examination, including measurement of axillary temperature and preparation of thick and thin blood smears for subsequent reference laboratory evaluation, on every patient interviewed. If a patient reported fever, or if the surveyor-measured temperature was ≥37.5 °C and the patient had not been prescribed first-line anti-malarials by the HW, a malaria RDT (SD Bioline malaria Pf^®^, Standard Diagnostics, Inc, Giheng-ku, Republic of Korea) was performed by the survey team and, if positive, the first-line anti-malarial was dispensed to the patient.

At the end of the day, all HWs conducting clinical consultations in the selected OPD on the day of the survey were interviewed. They were asked about their professional training, training in malaria case management, supervision, years of clinical experience, and if they had access to a copy of the latest malaria treatment guidelines dated July 2013. A visual confirmation of the available malaria treatment guidelines was made. Finally, HF assessments were completed for each site. Survey teams asked the facility in-charge about staffing and equipment, and visually verified the presence of malaria RDTs, drug stocks, drinking water for directly observed treatment, and other equipment related to malaria case management on the day of the study.

### Malaria case definitions

Malaria case definitions were based on the 2013 Malawi national treatment guidelines [9] (Table 1). Confirmed malaria was defined as all suspect uncomplicated malaria patients who received malaria testing at the HF (as part of routine case management) by either microscopy or RDT and tested positive prior to interview/enrolment in the study. Presumed malaria was defined as all suspect uncomplicated malaria patients who attended HFs with no malaria diagnostic services available on the day of the survey. Non-malaria was defined as all suspect uncomplicated malaria patients who attended HFs with malaria diagnostic services and tested negative by either microscopy or RDT at the health facility, prior to interview/enrolment in the study.

**Table 1:** Malaria definitions

1. Suspect uncomplicated malaria categories by age and pregnancy status
Children <5 years of age or pregnant women in the first trimester:
History of fever^a^ or measured axillary temperature ≥37.5 °C
Patients ≥5 years of age
History of fever^a^ or measured axillary temperature ≥37.5 °C AND at least one additional sign or symptom suggestive of malaria (i.e., chills, muscle or joint pain, headache, vomiting, diarrhoea, weakness, nausea, dizziness, fatigue or abdominal pain)
2. Suspect severe malaria
Patients with any of the following: history of convulsions, lethargy, no urine output, jaundice, coca-cola-coloured urine, palmar pallor, or vomiting everything
For patients <5 years of age, neck stiffness or unable to drink or breastfeed were also included

^a^History of fever was defined as: (1) Patient mentioned that their current illness involved a fever when asked by surveyor during the exit interview. (2) Patient spontaneously reported fever to the HW. (3) Patient reported fever to the HW or surveyor when asked

### Definition of outcomes

Outcomes were based on Malawi’s 2013 malaria treatment guidelines. Appropriate testing was defined as: (1) the patient presented with symptoms of suspect uncomplicated malaria (see Table 1); (2) at a facility with malaria-testing services available the day of the survey; and, (3) the patient was tested for malaria by either RDT or microscopy. Appropriate treatment was defined according to the following categories for suspect uncomplicated malaria patients: (1) attended HFs with malaria tests available, tested negative for malaria and did not receive an ACT; or (2) tested positive and received an ACT; or, (3) attended HFs without malaria tests available and received an ACT (presumptively treated for malaria). Patients were considered to have received an ACT if it had been prescribed, administered or dispensed at the HF prior to interview/enrollment in the study (to distinguish from treatments the study team initiated). Suspect severe malaria patients (see Table 1) were considered to have received appropriate treatment if they received intramuscular (IM) artesunate or IM quinine. In-patient intravenous treatment with artesunate at a larger hospital level should then follow.

### Statistical analyses

Data were analysed in SAS 9.3 (SAS Institute, Inc, Cary, NC, USA) and included weighted descriptive analyses of patient-level attributes adjusting for non-response and the sampling scheme, including clustering at the HF level. In addition unweighted descriptive analyses of HW and HF attributes were performed.

To explore factors associated with appropriate testing and treatment, univariable and multivariable logistic regression modelling was conducted for each outcome, using the SURVEYLOGISTIC procedure, which accounts for the weights and complex survey design, including clustering at the HF level [12, 13]. For the multivariable analysis, plausible effect modifiers were explored using 12 interaction terms and included in the model if significant. Variables were explored as interaction terms if there was plausibility for interaction or if they had been reported as such in peer-reviewed literature. Factors were included in the multivariable model if they had a *p* < 0.1 in the univariable analysis. The base multivariable model was checked for collinearity and confounding. Covariates were retained in the model as confounders if the odds ratio (OR) of any variable in the base model changed by at least 20%, unless the original variables in the base model had p > 0.05 and remained non-significant after adding potential confounders.

Factors associated with appropriate testing were examined for all suspect uncomplicated malaria patients attending HFs able to test for malaria on the day of the survey visit. However, analysis of factors related to appropriate treatment was limited to presumed uncomplicated malaria patients given the lack of variability (consistently high performance) for patients with positive diagnostic tests (confirmed malaria) and negative tests (not malaria). Considering the relatively small sample size of presumed malaria patients (n = 178), the most stable model with the lowest condition index [14] was selected after adding different combinations of patient-, HW- and HF-level factors.

## Results

### Descriptive summary

**Fig. 1:**
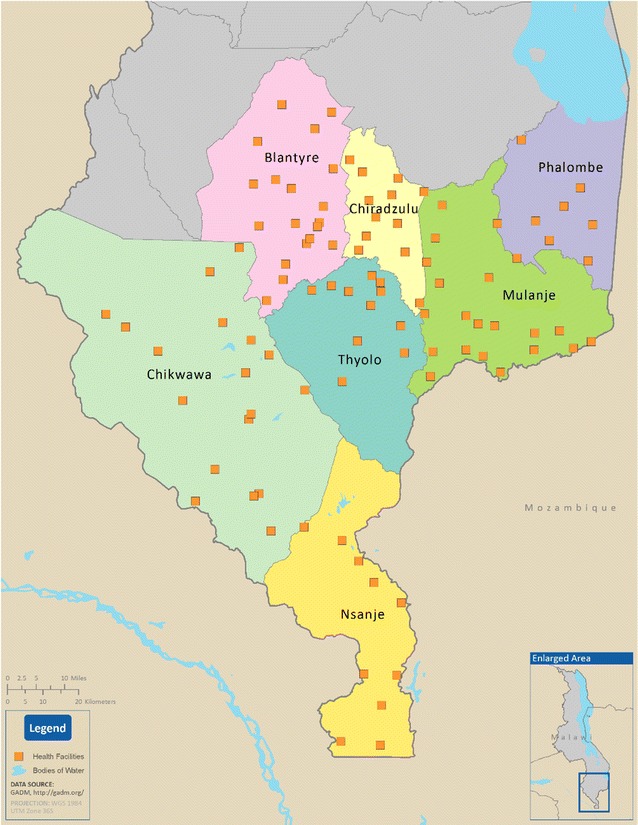
Map of southern Malawi showing 99 of 105 participating health facilities with GPS coordinates

The geographic distribution of the 105 facilities surveyed across the seven study districts is provided in Fig. 1. Altogether 153 HWs providing outpatient consultations at participating HFs during the survey period were enrolled, and interviews were completed with 150. A total of 2877 patients waiting in OPDs were screened and 2645 were eligible. Of these, 2567 (97.1%) consented to participate in the study and 2354 (89.0%) completed exit interviews. Patients were not interviewed if they were found to be too ill to complete the exit interview or were missed by the study team after they completed their clinical encounters.

### Health facility characteristics

Of the 105 surveyed HFs, 80% were operated by MoH (Table 2). The sample included 90 health centres, nine community hospitals and six district hospitals. Most HFs had functional malaria diagnostic services, primarily RDTs (79%), and only 25% had microscopy. Testing was usually available for the full day of the survey [94/105 facilities (90%)], but seven facilities did not have testing available the day of the survey and an additional four facilities had testing available for part of the survey day. Nearly half (48%) of HFs had a copy of 2013 national malaria treatment guidelines or other MoH reference material with malaria treatment content, for example, a primary care handbook.

**Table 2:** Summary characteristics of surveyed outpatient health facilities—Southern Malawi, 2015 (N = 105)

	n	%
Operation of health facility		
Ministry of Health	84	80.0
CHAM	21	20.0
Health facility type		
Health centre	90	85.7
Community hospital	9	8.6
District hospital	6	5.7
Diagnostic capacity		
Microscopy service functional for full day of survey	26	24.8
RDTs in stock for full day of survey	83	79.1
Either microscopy or RDT functional for day of survey^a^	94	89.5
Haemoglobin testing functional for full day	11	10.5
Infrastructure and equipment available on day of survey		
Clean drinking water	93	88.6
Cups for administering oral medications available	69	65.7
Functioning hanging or standing scale	98	90.5
Functioning thermometer	69	65.7
Availability of 2013 malaria treatment guidelines on day of survey		
Copy of 2013 national or other reference material on malaria treatment guidelines	50	47.6
Wall flowchart with 2013 malaria treatment guidelines	14	13.3

Anti-malarial drugs in stock for the full day on day of survey		
A. First-line treatment for uncomplicated malaria in pregnant women (1st trimester) and children <5 kg		
Quinine tablets	37	35.2
B. First-line treatment for uncomplicated malaria in all other patients		
At least one artemether-lumefantrine (AL) formulation	96	91.4
AL 1 × 6 dose-pack (regular or dispersible)	88	83.8
AL 2 × 6 dose-pack (regular or dispersible)	77	73.3
AL 3 × 6 dose-pack	83	79.1
AL 4 × 6 dose-pack	77	73.3
C. Second-line treatment for uncomplicated malaria (treatment failure or when AL is contra-indicated)		
Artesunate-amodiaquine (co-formulated or co-blistered)	32	30.5
Any first- or second-line anti-malarial treatment for uncomplicated malaria	98	93.3
D. Pre-referral treatment for severe malaria		
Any injectable pre-referral treatment (artesunate or quinine)	103	98.1
Parenteral artesunate	88	83.8
Parenteral quinine	94	89.5
Artesunate suppositories	6	5.7

^a^An additional 4 facilities had testing availability for part of the day

Ninety-six (91%) HFs had either the first-line (AL) or second-line (artesunate-amodiaquine (ASAQ)) ACT available for the full day of the survey. The majority of HFs (91%) had at least one AL formulation available, with individual dose-pack availability ranging from 73% for both AL 2 × 6 and AL 4 × 6 dose-packs, 79% for AL 3 × 6, to 84% for AL 1 × 6 dose-packs. ASAQ was in stock at 31% of HFs. Quinine tablets were in stock at 35%, parenteral artesunate at 84% and parenteral quinine at 89% of HFs. Nearly all HFs (98%) had at least one injectable pre-referral treatment in stock.

### Health worker characteristics

Of 150 HWs performing outpatient consultations during the survey, 73% were male; median HW age was 29 years, with a median of four years of clinical experience (Table 3). Nearly half (47%) had a copy of the 2013 national malaria treatment guidelines, and 67% had received at least one supervision visit in the previous 6 months.

**Table 3:** Summary characteristics of health workers providing outpatient care at surveyed health facilities—Southern Malawi, 2015 (N = 150^a^)

	n	%
Cadre of health worker caring for patients in sampled outpatient department		
Medical assistant (2 years of formal training plus 1 year internship)	100	66.7
Clinical officer (3 years of formal training plus 1 year internship)	23	15.3
Nurse^b^ (at least 3 years of formal training)	21	14.0
Patient attendant (no formal training)	4	2.7
Pharmacy technician (2 years of formal training)	1	0.7
Health surveillance assistant (6 weeks of formal training)	1	0.7
Training and guidelines		
Malaria case management (in-service)^c^ training ≥2013	113	75.3
Malaria case management (on-the-job)^d^ training ≥2013	53	35.3
Either in-service or on-the-job malaria case management training ≥2013	128	85.3
Integrated management of childhood illness (IMCI) training within the last 5 years	27	18.0
Has a copy of the latest malaria treatment guidelines (dated ≥2013)	70	46.7
Supervision		
Received any supervision in the past 6 months	101	67.3
Received at least two supervisory visits in the past 6 months	67	44.7
Supervision visits that included observation of patient consultations	55	36.7
At least one supervisory visit with observation of patient consultations that involved assessing health workers’ knowledge and prescription practices of anti-malarial drugs^e^	36	24.0
Other characteristics	Median (IQR)	Min–Max
Age	29.0 (26.0, 36.0)	21–75
Years of experience	4.0 (2.0, 7.0)	0–45

^a^Six of the 150 HWs did not see any enrolled patients but were interviewed by the survey teams

^b^Nurse category includes 20 nurse midwife technicians (3 years of formal training) and a Registered nurse (with a 4-year degree)

^c^In-service malaria case-management training refers to a formal, typically off-site multiday training on malaria diagnosis and treatment

^d^On-the-job training is an informal training provided to health workers at their place of employment by the facility in-charge, a co-worker, NGO staff, District Health Management Team or other Ministry of Health staff

^e^Assessment of health workers’ knowledge on malaria treatment practices involves any or all of the following: (1) direct in-office observation of patient consultations; (2) review of patients’ health passports for malaria laboratory results and prescriptions; (3) quizzing HWs on national anti-malarial treatment guidelines; (4) reviewing dosing schedule of prescribed malaria prescriptions with patients to evaluate their understanding

### Patient characteristics

For the 2342 patients who completed the exit interview, 723 (28%) were under 5 years old; among these younger patients, 79% had suspect uncomplicated malaria and 6% had suspect severe malaria (Table 4). Overall, 73% of all study participants had suspect malaria, the majority of whom (95%) had suspect uncomplicated malaria. Of 1619 patients aged at least 5 years, 67% had suspect uncomplicated malaria and 2% had suspect severe malaria. In this study, 69% of suspect malaria patients spontaneously reported fever to the HW (Table 4). When patients did not spontaneously report fever, HWs asked patients about it a little over half the time (52%). Other symptoms spontaneously reported to the HW included headache (33%), vomiting (17%) and skin problem (6% of suspect malaria patients).

**Table 4:** Characteristics and outcomes for patients who completed the exit interview—Southern Malawi, 2015

	n/N	Weighted percent (95% CI)
Demographics (years)		
<5	723/2342	28.3 (21.8, 34.8)
≥5	1619/2342	71.7 (65.2, 78.2)
Suspect malaria classification		
Suspect malaria cases (both uncomplicated and severe)	1695/2342	73.4 (70.2, 76.6)
Suspect uncomplicated malaria	1605/1695	95.4 (94.2, 96.6)
Suspect severe malaria	90/1695	4.6 (3.4, 5.8)
Symptoms spontaneously reported to HW by suspect malaria patients		
Fever	1146/1695	68.5 (64.0, 73.0)
When patient did not spontaneously report fever, HW asked about fever	295/549	51.6 (46.0, 57.2)
Headache	553/1695	33.4 (28.6, 38.2)
Vomiting	284/1695	16.9 (14.3, 19.5)
Skin problem	99/1695	5.5 (3.9, 6.9)
Testing for suspect uncomplicated malaria patients who attended HFs with diagnostic tests		
Tested by either microscopy or RDT	1072/1427	75.7 (68.9, 82.5)
Positive RDT or microscopy	547/1072	53.2 (46.0, 60.4)
Medications administered, prescribed or dispensed to confirmed uncomplicated malaria patients		
Treated with first- or second-line antimalarial (AM)^a^	511/547	92.7 (85.4, 99.9)
Treated with other anti-malarial^b^	12/547	4.7 (0.0, 11.9)
No anti-malarial	24/547	2.6 (1.2, 4.0)
Medications administered, prescribed or dispensed to presumed uncomplicated malaria patients		
Treated with first- or second-line AM^a^	98/178	60.3 (45.1, 75.5)
Treated with other anti-malarial^c^	5/178	2.8 (0.0, 8.3)
No anti-malarial	75/178	36.8 (22.8, 50.8)
Suspect severe malaria		
Received recommended pre-referral anti-malarial^d^	8/90	5.7 (0.3, 11.0)
Referred or told to get in-patient admission right away	11/90	6.9 (1.9, 11.9)
Received pre-referral anti-malarial *plus* immediate referral/admission	3/90	1.6 (0.0, 3.6)
Treated with other anti-malarial^e^	46/90	59.6 (46.4, 72.8)
No anti-malarial	36/90	34.7 (22.4, 47.0)

^a^First- or second-line AM refers to artemether-lumefantrine (AL) or artesunate-amodiaquine (ASAQ) for all except pregnant women in first trimester and children weighing <5 kg who get quinine. Numerator includes eight confirmed malaria patients who got both AL and intramuscular (IM) quinine and one confirmed malaria patient who got both AL and IM artesunate

^b^Oral quinine (n = 6), IM quinine (n = 3), AL to women in first trimester (n = 2), SP (n = 1)

^c^All given sulfadoxine-pyrimethamine (SP) none of whom was pregnant

^d^Three received IM artesunate, five received IM quinine

^e^42 patients received only AL, one received both AL and SP, one received ASAQ and two received oral quinine

### Malaria testing and treatment practices among suspect malaria patients

Of 2096 patients who attended facilities with malaria testing services, 1427 (70%) had suspect uncomplicated malaria and therefore required parasitologic testing. Of those, 76% were tested, over half (53%) of whom tested positive (confirmed malaria). Although most confirmed malaria patients (93%) were appropriately treated, nearly 5% received a non-recommended anti-malarial (quinine, sulfadoxine-pyrimethamine (SP), or AL for pregnant women in the first trimester), and 3% received no anti-malarial. Of 525 patients with negative test results, 96% were not treated with any anti-malarial. Among the 29 patients who received an anti-malarial, 25 received AL, two received quinine, and two received SP (none was pregnant).

Of 246 patients who attended facilities without malaria testing services, 75% had presumed uncomplicated malaria. The first- or second-line anti-malarial was prescribed, administered or dispensed to 60% of them, 3% received SP (none was pregnant), and 37% received no anti-malarial.

**Fig. 2:**
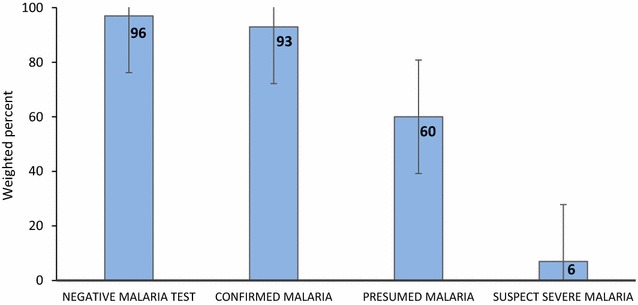
Summary of appropriate malaria treatment practices among suspect malaria patients

Of 90 suspect severe malaria patients, 53% had a sign of severe malaria documented in their record by the HW. However, only 6% of the 90 patients received appropriate pre-referral treatment, 7% were immediately referred to the hospital or admitted, and only 2% received both pre-referral treatment and referral/admission, the recommended treatment for severe malaria according to the national malaria treatment guidelines. Most (60%) suspect severe malaria patients were given an oral anti-malarial, mainly AL; 35% did not receive any anti-malarial. A summary of appropriate malaria treatment practices is provided in Fig. 2. In accordance with the guidelines, malaria testing was not considered as part of appropriate treatment for suspect severe malaria.

### Dispensing and dosing practices and patient knowledge among uncomplicated malaria patients who had an ACT dispensed at the HF

Of 609 uncomplicated malaria patients who received an ACT (either AL or ASAQ), 534 (89%) had the drug dispensed at the HF (Table 5); dosing was correct for 95%. Correct dosing was based on age and weight as stated in the guidelines. While 92% of uncomplicated malaria patients received an explanation from the HW regarding how to take the ACT at home, only 8% were told what to do in case of vomiting within 30 min of taking the drug.

**Table 5:** Artemisinin combination therapy (artemether-lumefantrine or artesunate-amodiaquine) dispensing, dosing and patient knowledge for uncomplicated malaria patients

	n/N	Weighted percent (95% CI)
*1. ACT dispensing and dosing*		
Total number of uncomplicated (confirmed and presumed) malaria patients	725/2342	33.0 (27.2, 38.8)
Received an ACT (AL or ASAQ)^a^	609/725	84.9 (77.2, 92.5)
ACT dispensed	534/609	88.9 (80.1, 97.7)
ACT correctly dosed when dispensed^b^	505/534	94.9 (92.7, 97.2)
Type of ACT dispensed		
AL dispensed	517/534	97.2 (93.3, 100.0)
AL correctly dosed when dispensed	490/534	92.5 (88.2, 96.8)
ASAQ dispensed	17/534	2.8 (0.0, 6.7)
ASAQ correctly dosed when dispensed^c^	16/17	93.6 (3.7, 100.0)
HWs provided patients with the following ACT counselling information		
First dose of ACT given at HF	72/534	16.8 (3.2, 30.3)
HW explained how to take ACT at home	495/534	91.8 (88.7, 94.8)
HW advised what to do in case of vomiting within 30 min of taking ACT	36/534	7.5 (4.8, 10.1)
HW instructed to complete all doses of ACT even if he/she feels better	316/534	60.9 (52.7, 69.1)
HW instructed to take AL with food, milk or milk containing drink^d^	141/517	27.9 (20.2, 35.7)
*2. Patient ACT dosing knowledge*		
Correct knowledge regarding all aspects of ACT dosing^e^	404/534	78.1 (72.3, 83.8)
Knows correct amount of ACT (tablet or syrup) to take per dose	460/534	85.9 (81.3, 90.6)
Knows total number of days to complete ACT dose	473/534	90.6 (86.8, 94.3)
Knows correct number of AL doses to take per day	474/534	89.5 (86.0, 93.0)

^a^1 confirmed malaria patient had both AL and ASAQ dispensed; AL was the only ACT dispensed to presumed malaria patients

^b^ASAQ dosing information was coded as correct for patients who received the correct strength or number of ASAQ tablets and for those with missing dosing information

^c^1 patient (a 5 years old child weighing 14 kg) received lower than recommended strength formulation for ASAQ

^d^Only pertains to patients who were dispensed AL. Patients with ASAQ were not asked this question

^e^Aspects of AL dosing were evaluated when the drug was dispensed and included patient knowledge of correct: number of AL tablets to take at each dose, number of AL doses to take per day and number of days to complete the AL dose. Aspects of correct ASAQ dosing included patient knowledge of correct number of ASAQ tablets to each for each dose and the total number of days to complete the entire ASAQ dose

Most patients with uncomplicated malaria (78%) demonstrated correct knowledge regarding AL dosing, including the amount of drug per dose, the number of doses per day and the total number of days to complete the full dose for patients who received AL (Table 5).

### Factors associated with appropriate testing among suspect uncomplicated malaria patients

Fourteen patient-level, ten HW-level, six HF-level factors, and 12 interaction terms were tested for association with HW testing practices among suspect uncomplicated malaria patients in univariable logistic regression. None of the HW or HF factors was included in the multivariable logistic model model, as they had *p* > 0.1 in univariable analyses, but were tested for confounding. None of them was found to be confounders. All patient-level factors included in the multivariable model were statistically significant (Table 6). The model also included an interaction between a HW receiving at least one supervision visit in the previous 6 months and possession of a copy of the 2013 national malaria treatment guidelines. Of 1427 suspect uncomplicated malaria patients who should have been tested for malaria, those who spontaneously reported history of fever (OR 2.6; 95% CI 1.7–4.0), headache (OR 1.5; 95% CI 1.1–2.2), and vomiting to the HW had higher odds of getting tested (OR 2.0; 95% CI 1.0–4.0). Conversely, patients who spontaneously reported a skin problem to the HW were 60% less likely to get tested (OR 0.4; 95% CI 0.2–0.6).

**Table 6:** Factors associated with appropriate testing among suspect uncomplicated malaria patients (N = 1427)

Factors	Number of patients	Number tested (weighted %)	Unadjusted OR (95% CI)	*p* value	Adjusted OR (95% CI)	*p* value
Patient spontaneously reported these complaints to the HW						
Fever						
Yes	950	784 (81.8)	2.6 (1.6–4.3)	<0.0001	2.6 (1.7–4.0)	<0.0001
No	477	288 (62.9)	Ref		Ref	
Headache						
Yes	481	380 (79.4)	1.4 (0.9–1.9)	0.055	1.5 (1.1–2.2)	0.017
No	946	692 (73.8)	Ref		Ref	
Vomiting						
Yes	223	194 (86.1)	2.2 (1.2–4.2)	0.016	2.0 (1.0–4.0)	0.040
No	1204	878 (73.8)	Ref		Ref	
Skin problem						
Yes	80	37 (49.0)	0.3 (0.2–0.5)	<0.0001	0.4 (0.2–0.6)	0.0001
No	1347	1035 (77.1)	Ref		Ref	

	n/N	Weighted percent (95% CI)	OR (95% CI)	p value
Interaction term included in the model: supervision * 2013 malaria treatment guidelines available				
Supervision * Guidelines	299/389	75.9 (66.1, 85.8)	2.2 (0.8, 6.6)	0.143
Supervision * No guidelines	438/559	78.8 (69.5, 88.0)	2.7 (0.9, 8.0)	0.073
No supervision * Guidelines	161/190	87.4 (77.6, 97.2)	6.2 (1.6, 24.7)	0.009
No supervision * No guidelines	174/289	56.8 (36.2, 77.4)	Ref	

Ref means referent group

Interaction term included in the model. Other factors with p value ≥0.1 in the univariate analysis that were not included in the multivariable model were: (1) Patient-level factors: patient spontaneous report to the HW of malaria, chills, fatigue, joint pain, weakness or cough; (2) HW-level factors: HW age, number of patients seen by the HW, number of years of formal training received, number of years of clinical experience, latest training on malaria case management, access to the latest malaria treatment guidelines and supervision in the last 6 months; (3) HF-level factors: MoH vs CHAM operated HFs, availability of thermometers, number of patients at the HF and the number of HWs at the HF

Interestingly, the effect of having access to a copy of the 2013 national malaria treatment guidelines on testing differed depending on whether or not the HW had had a supervision visit in the previous 6 months (Table 6). For HWs that were not supervised, the odds of testing was higher among those who had guidelines compared to those who did not. Only 57% of patients seen by HWs who were neither supervised in the previous 6 months nor possessed a copy of the guidelines were tested, compared to 76, 79 and 87% of patients seen by HWs with both supervision and guidelines, supervision but not guidelines, and guidelines but not supervision, respectively.

Of the 289 suspect uncomplicated malaria patients seen by HWs with neither supervision nor guidelines, 46 were seen by HWs whose colleagues at the facility had received some supervision or guidelines and 76% of them were tested for malaria (same testing rate for the rest of the study population). The other 243 patients were seen by HWs who either: (1) worked alone and had received neither supervision nor possessed a copy of the guidelines; or, (2) worked with other HWs who like themselves, had received neither. Only 52% of patients seen by this latter category of HWs were tested. In total, there were 18 HWs located at 16 HFs in this category. There were no observed differences between these 18 HWs and other HWs in terms of age, years of clinical experience, type of formal training, malaria-related training received, or the type of HF where they worked. Furthermore, there were no statistically significant differences in distances of these HFs where HWs had neither recent supervision nor guidelines to their respective district hospitals than between the other facilities and the district hospitals.

### Factors associated with appropriate treatment among presumed uncomplicated malaria patients

The same patient-, HW- and HF-level factors and 12 interaction terms were evaluated for an association with correct treatment among presumed uncomplicated malaria patients. Patients who spontaneously reported history of fever to the HW had a nearly six-fold higher odds of appropriate treatment (OR 5.7; 95% CI 1.9–17.6) (Table 7). The odds of appropriate malaria treatment were also increased as measured temperature increased (for each degree rise in patient temperature OR 1.5; 95% CI 1.1–1.9), HW age increased (for each additional year in HW age OR 1.1; 95% CI 1.0–1.1), and number of supervision visits in the previous 6 months to the HW increased (OR 1.2; 95% CI 1.0–1.4). A patient’s spontaneous complaint of headache to the HW was a confounder and was therefore included in the final model. Notably, for both the models of correct testing and treatment, there was no significant association with HWs who had received the latest training on malaria case management, whether they had a copy of the latest malaria testing guidelines, or their length of clinical experience.

**Table 7:** Factors associated with appropriate treatment among presumed uncomplicated malaria patients^a^ (N = 178)

Factors	Number of patients	Number treated (weighted %)	Unadjusted OR (95% CI)	*p* value	Adjusted OR (95% CI)	*p* value
Patient spontaneously reported fever to the HW						
Yes	126	79 (68.6)	3.5 (1.7–7.3)	0.001	5.7 (1.9–17.6)	0.002
No	52	19 (38.4)	Ref		Ref	
Patient temperature (°C)—surveyor measured			1.3 (1.0–1.7)	0.032	1.5 (1.1–1.9)	0.005
HW age (years)			1.0 (1.0–1.1)	0.001	1.1 (1.0–1.1)	<0.0001
Number of supervision visits			1.1 (0.9–1.3)	0.135	1.2 (1.0–1.4)	0.029

Data from facilities unable to test patients at the time of the survey

^a^Only statistically significant factors with p < 0.05 in the multivariable analysis have odds ratio estimates shown in the table above. Other factors included in the logistic regression model were: patient age in years, patient spontaneous complaint of cough and headache (which was a confounder). Other factors with p value ≥0.1 in the univariate analysis that were not included in the multivariable model were: (1) Patient-level factors: patient spontaneous report of malaria, vomiting, chills, fatigue, joint pain, skin problem, weakness or cough to the HW; (2) HW-level factors: Number of patients seen by the HW, number of years of formal training received, number of years of clinical experience, latest training on malaria case management, and access to the latest malaria treatment guidelines; (3) HF-level factors: MoH vs CHAM operated HFs, availability of thermometers, number of patients at the HF and the number of HWs at the HF

## Discussion

Malaria case-management services, including both diagnostics and treatment, were widely available in outpatient HFs in southern Malawi, representing tremendous progress over the past five years. A national health facility survey in 2011, before RDTs became widely available, found that only 24% of HFs across the country had functional microscopy services and 81% had at least one AL dose-pack in stock [7], while in this study 90% had diagnostic services and 91% had at least one formulation of AL available. The majority of HWs were adherent to the national malaria treatment guidelines when testing for malaria, evidenced by the 76% of suspect uncomplicated malaria patients who were tested for malaria when tests were available. This proportion is similar to that reported in Kenya when both microscopy and RDT were available at HFs [15] but higher than that reported in many other malaria high-transmission settings since release of the WHO recommendation for universal access to malaria diagnostic testing [16–22]. Most testing was done using RDTs, likely because they were more widely available on the day of the survey, and microscopy is limited to higher-level facilities in Malawi. The widespread use of RDTs reflects their acceptability and consistent supply as a malaria diagnostic tool since they were introduced in Malawi in 2011.

HWs were more likely to test for malaria when patients spontaneously reported fever, vomiting or headache, and were less likely to test when patients reported a skin problem. A similar association between patient-reported symptoms of fever and skin problem on malaria treatment has been reported in Uganda [19]; in Vanuatu, patients with a main complaint of fever were more likely to get tested for malaria [20].

Similar to other studies in Malawi, Central African Republic and Zambia, neither supervision nor guidelines alone were associated with HW practices [23–25]. In this study, analyses identified an interaction between HW supervision in the previous 6 months and access to the latest national malaria treatment guidelines. Unlike most interactions, this did not reflect synergy between these two factors but rather that association between guidelines and testing varied by whether or not the HW received supervision. It is difficult to explain this finding with information in this study, as HWs with neither supervision nor guidelines may possess characteristics that were not measured. A further assessment of these HWs may be needed to support them better, but immediate priority should be given to ensuring that these HWs have at least regular supervision and copies of guidelines.

The vast majority of patients were seen at facilities (91%) with malaria diagnostics available on the day of the survey. When these patients tested positive for malaria, nearly all (93%) received correct treatment for malaria. In contrast, when testing was not available and patients were presumed to have malaria, treatment was sub-optimal, with only 60% of presumed malaria patients receiving a recommended treatment. This suggests that HWs make better malaria treatment decisions based on diagnostic test results. Furthermore, when HWs treated patients for malaria, they followed guidelines in selecting the recommended first- or second-line anti-malarial most of the time. In addition, HWs generally accepted negative test results: only 3% of test-negative (either RDT or microscopy) patients received an anti-malarial. This is much lower than the 31% treatment rate reported in 2011 for microscopy-negative patients [8] and substantially lower than treatment rates for test-negative patients in studies from Kenya, Tanzania, Uganda, and Burkina Faso [15, 26–28].

Pre-referral treatment for suspect severe malaria was extremely poor. The majority of patients were not given IM artesunate or quinine despite the fact that HWs documented a sign or symptom of severe malaria for over half of them. This is especially concerning since most HWs in the study recently received malaria case management refresher training that focused on treatment of severe malaria. Interestingly, half (53%) of suspect severe malaria patients were given an oral ACT, which may reflect a lack of awareness of severe malaria symptoms or a perception of treatment guidelines as a proposition rather than a recommendation requiring strict adherence. It is also concerning that 35% of suspect severe malaria patients did not get any anti-malarial, as mortality from untreated severe malaria approaches 100% [29]. Pre-referral treatment for suspect severe malaria is critical for patients of all ages; adults >50 years have been reported to have a higher risk of death from severe malaria compared to children <10 years [30]. Encouragingly, a recent study found that when patients are admitted to hospitals with a diagnosis of severe malaria they had high rates of correct treatment at 93% [31]. However, a better understanding of why HWs rarely follow guidelines for managing severely ill patients at the outpatient level is needed.

Furthermore, when HWs did not have access to diagnostic testing, the odds of appropriate treatment increased nearly six-fold for patients who spontaneously reported history of fever to the HW. This finding was similar in Kenya [32]. Despite all patients with suspect malaria presenting with fever or history of fever, fever was spontaneously reported to HWs by only two-thirds of suspect uncomplicated malaria patients and only half of those who did not report fever had the HW ask about it. This highlights an opportunity for targeting malaria-related public health interventions at the patient-HW interaction, encouraging patients to report fever to HWs and reminding HWs to ask about fever. Unlike other studies which have found that lower-level HWs tend to adhere to guidelines better than higher-level cadres [19, 20, 32], an association between HW cadre and malaria testing or treatment was not identified in this study.

Vomiting is a common malaria symptom, therefore having patients understand that they should take another dose in case of vomiting within half-an-hour is important in treating malaria appropriately. Only 8% of patients were advised what to do in case of vomiting within 30 min of taking the ACT. In addition, only 17% of uncomplicated malaria patients who received the drug were given the first dose at the HF. This is a missed opportunity to promote adherence to dispensed medications. Patients have been reported to be more likely to complete all the prescribed doses if they are given the first dose at the HF [33]. Furthermore, opportunities exist in instructing patients to take AL with food and to complete all doses of the ACT even when patients feel better. This study did not explore why HWs miss these important counselling messages when dispensing ACT. With most HFs having access to clean drinking water and cups for administering oral medications on the day of the survey, it was expected that a higher proportion of patients would have taken the first dose at the HF. Perhaps HWs are not fully aware of the importance of directly observing patients taking the first dose and perhaps assume that patients will intuitively know to return to the HF for more ACT doses in case of vomiting within half-an-hour of taking the medication or the workload does not allow enough room for counseling. A thorough qualitative study to understand what drives health workers to provide counseling services may be needed to better understand this. Despite HWs not fully providing all necessary drug counselling messages, most patients expressed good understanding of the correct dosing regimen prescribed.

## Limitations

This study has several limitations. Survey teams did not directly observe patient-HW interactions and relied on patient report, which is subject to recall bias. However, over 95% of patients had their clinical encounter recorded in the health booklet, which was examined by the survey teams. The study was limited to seven districts in southern Malawi that had received malaria case management refresher training in 2014. The results of this study cannot be generalized to the entire nation of Malawi or to districts where HWs had not received the same malaria refresher training. However, HFs in this region are typical of other HFs across Malawi. It is therefore expected that the malaria case-management practices in this region are similar to those for the rest of the country, and previous studies have found no significant regional difference in malaria treatment practices [7]. The presence of survey teams at HFs likely influenced HW practices even though they did not directly observe their work. This may have overestimated appropriate treatment since HWs may have been more likely to follow guidelines under assumed observation. Furthermore, the multivariable analysis for appropriate treatment was limited to 178 presumed malaria patients seen at ten HFs, a small sample size that may have underpowered the analysis.

Finally, the accuracy and validity of RDT results was not evaluated to make sure that HWs followed manufacturer instructions and that the kits were in good condition.

## Conclusions

Malawi has shown tremendous progress in malaria case management since the ‘test-and-treat’ policy was implemented in HFs by the MoH. Most HFs have diagnostic test availability, and management of patients who test positive (confirmed malaria) or negative (not malaria) was excellent at 93 and 96%, respectively. However, there is much room for improving management of malaria, especially when testing is not available and when patient symptoms are severe. Targeting public health interventions at the patient-HW interaction to encourage patients to report history of fever to the HW and remind HWs to ask about fever or measure temperature is critical to improving both malaria testing and treatment practices in southern Malawi. In addition, the NMCP could provide extra supervision visits to HWs, offer more support to younger HWs, and eliminate RDT stock-outs by strengthening supply chain management. Finally, immediate attention should be given to improving HW recognition and pre-referral treatment of suspect severe malaria, which was vastly undertreated in this study.
